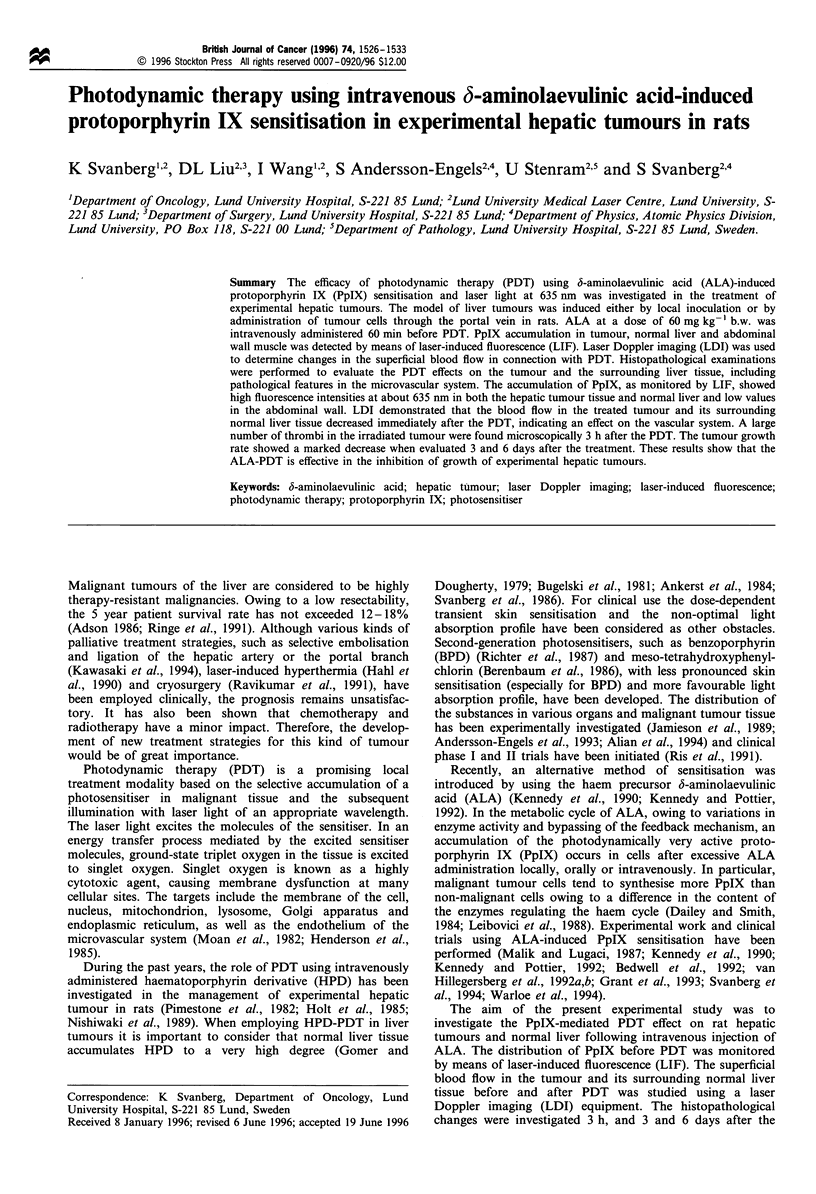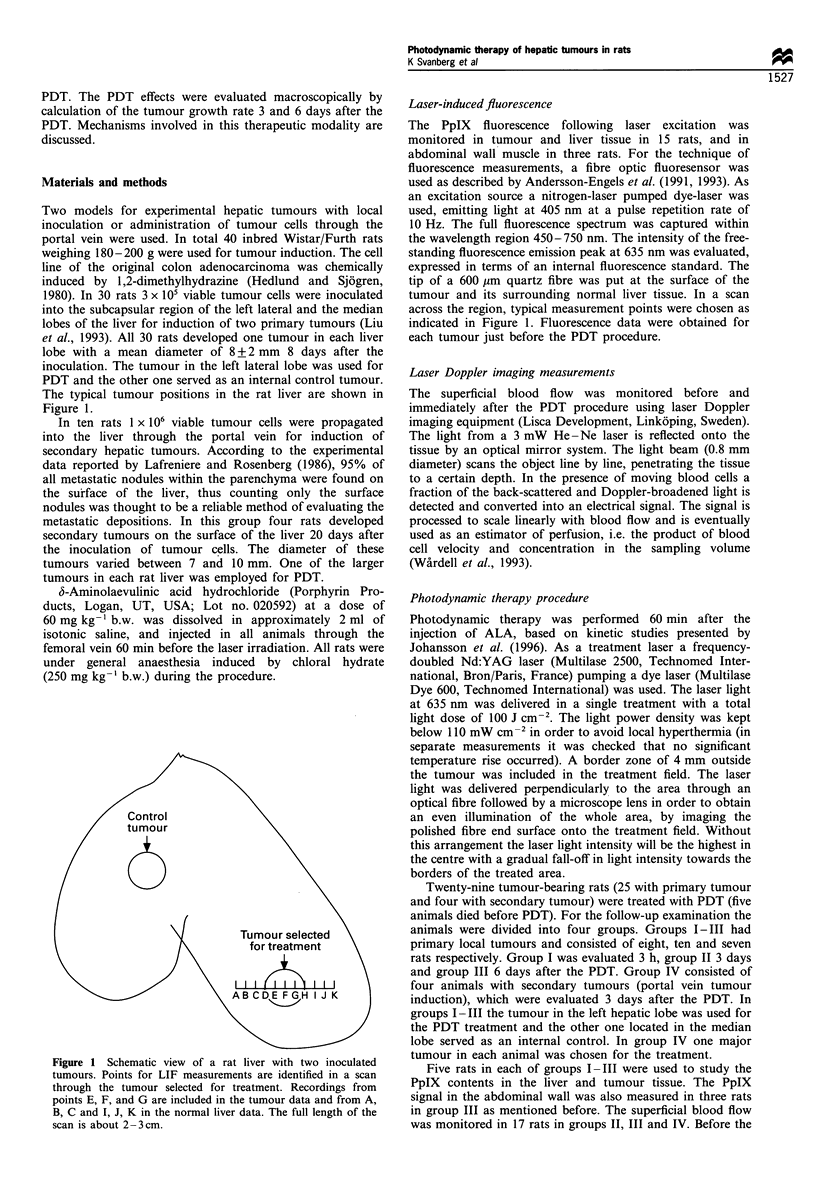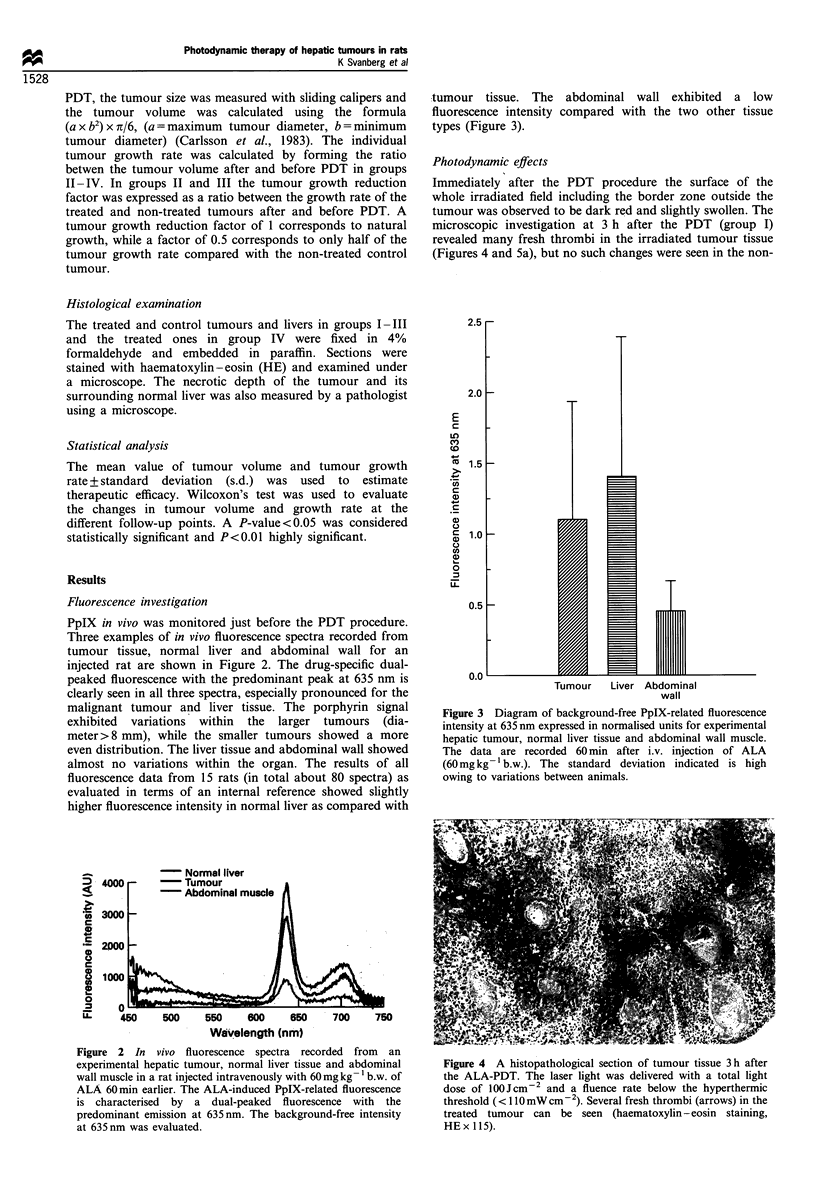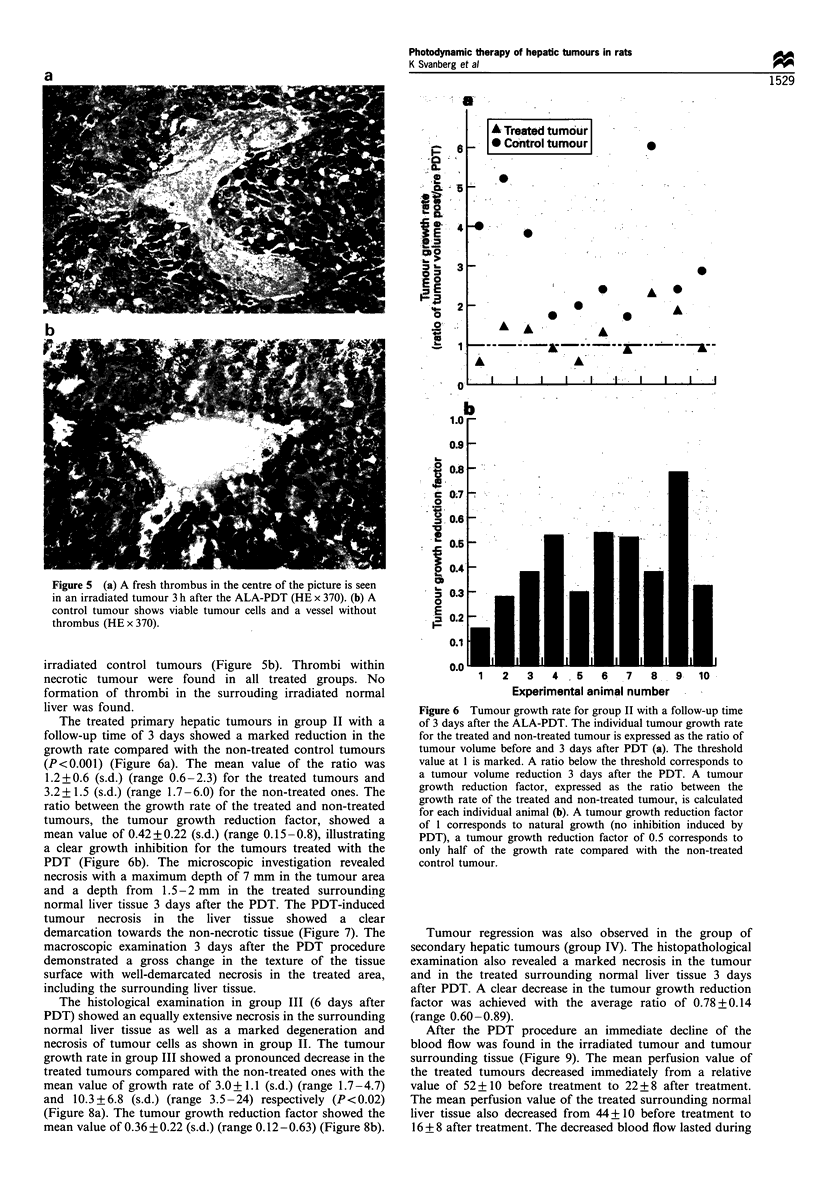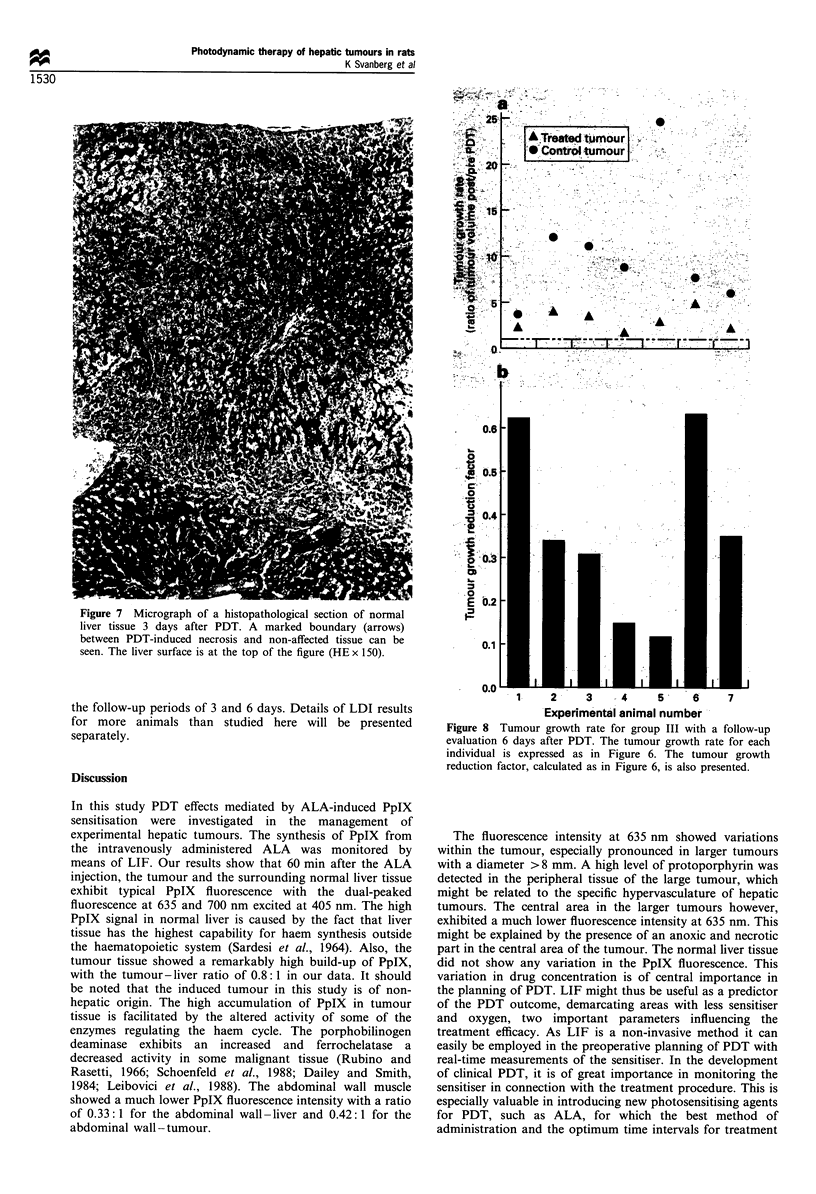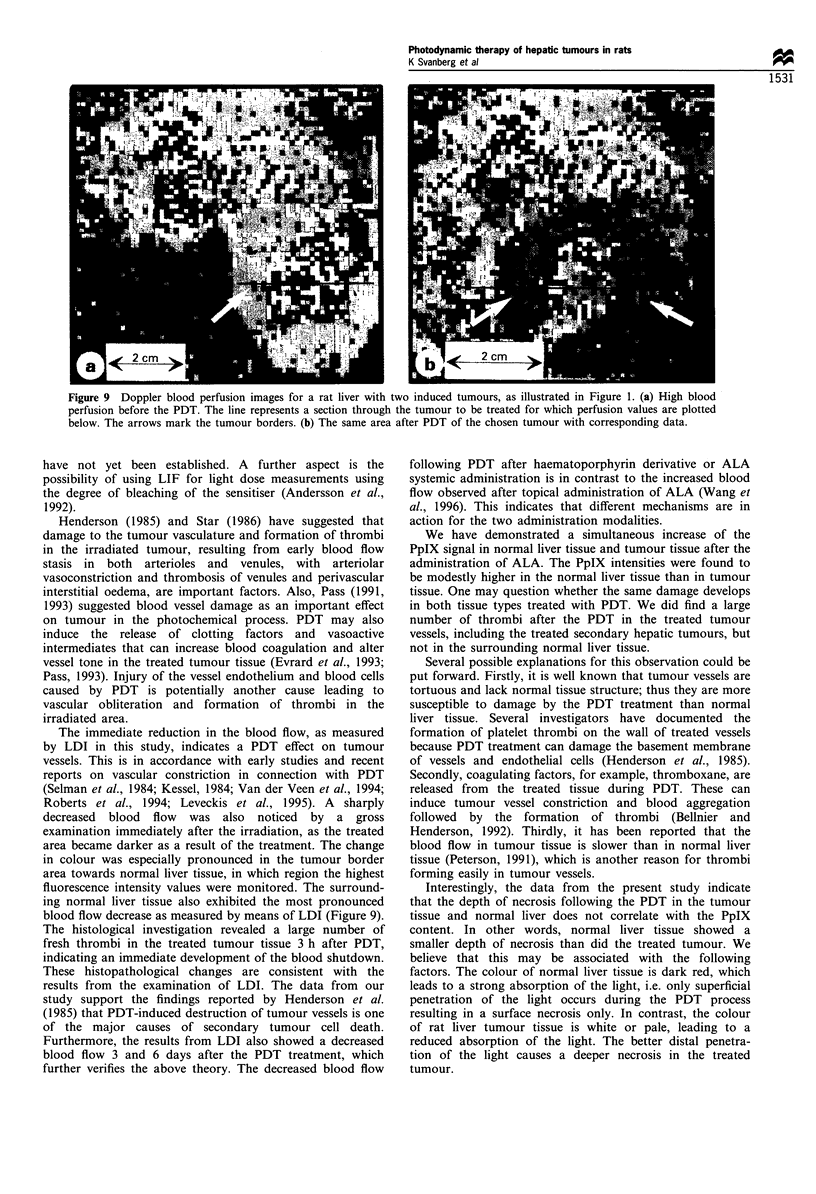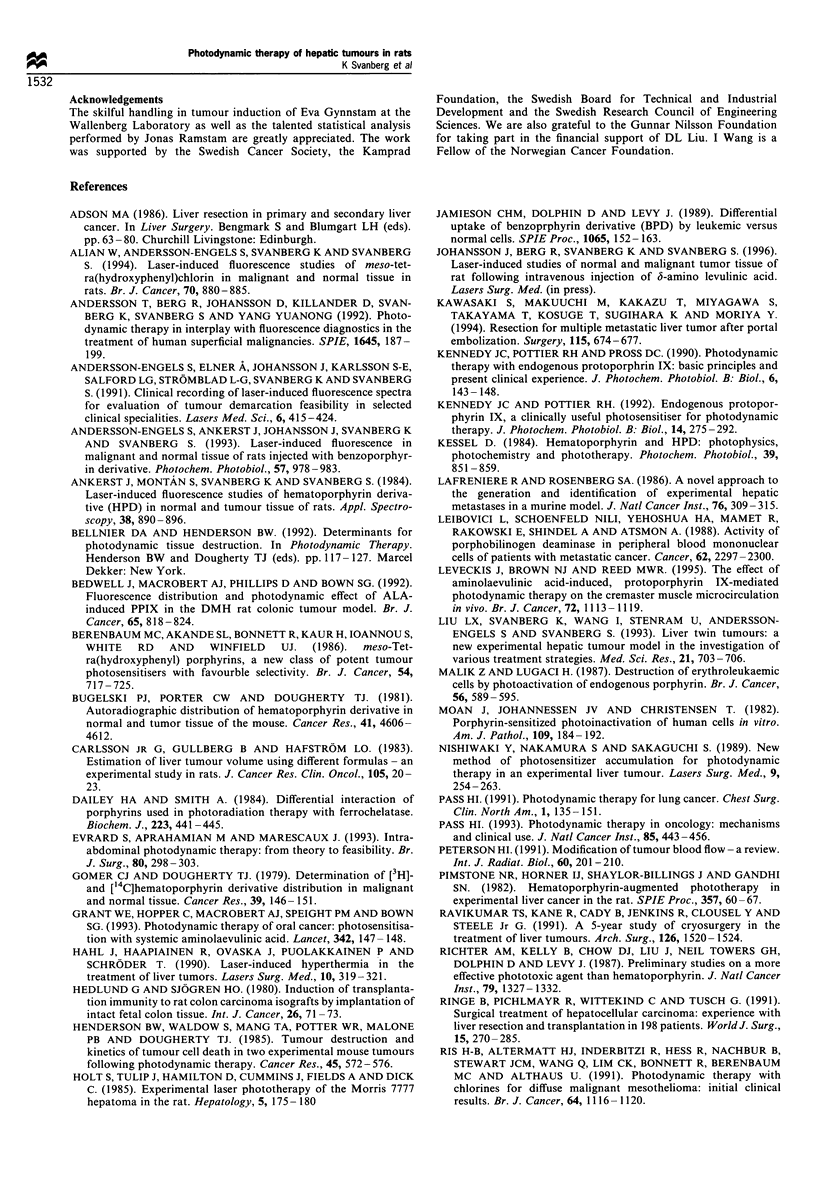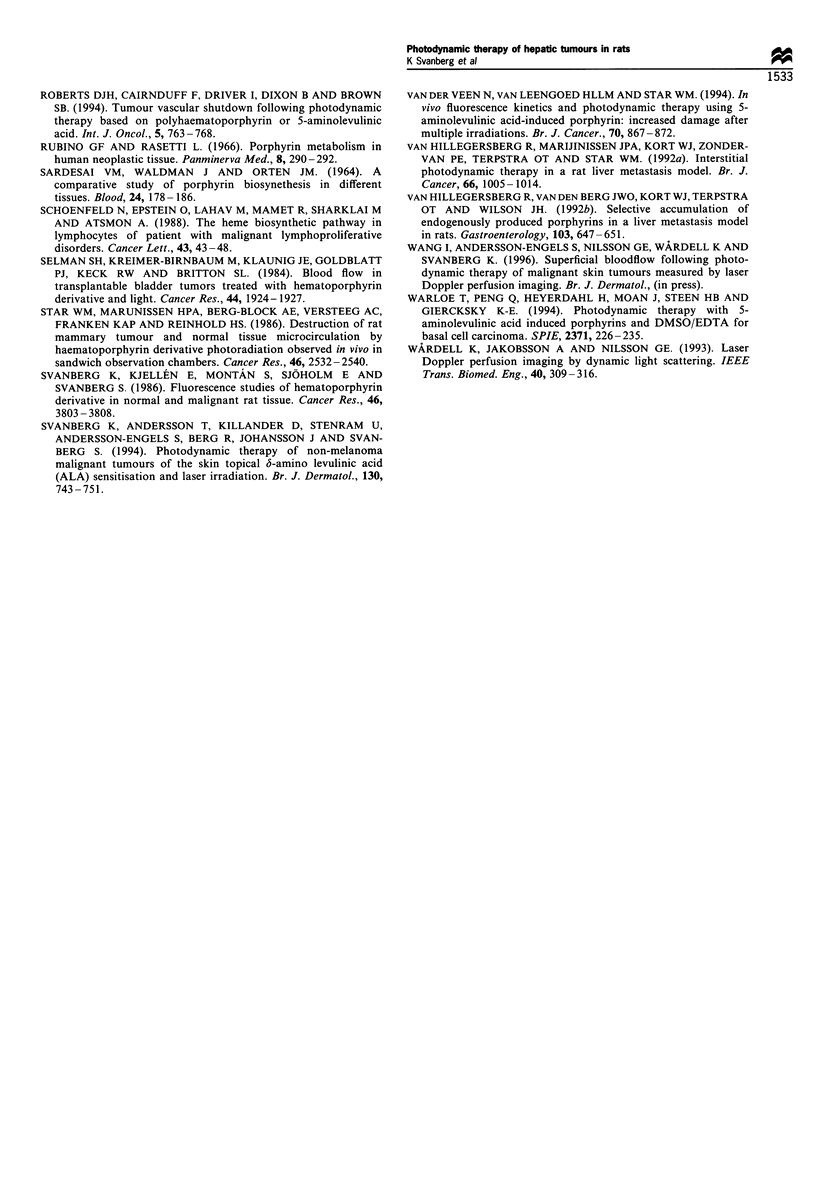# Photodynamic therapy using intravenous delta-aminolaevulinic acid-induced protoporphyrin IX sensitisation in experimental hepatic tumours in rats.

**DOI:** 10.1038/bjc.1996.584

**Published:** 1996-11

**Authors:** K. Svanberg, D. L. Liu, I. Wang, S. Andersson-Engels, U. Stenram, S. Svanberg

**Affiliations:** Department of Oncology, Lund University Hospital, Sweden.

## Abstract

**Images:**


British Journal of Cancer (1996) 74, 1526-1533
© 1996 Stockton Press All rights reserved 0007-0920/96 $12.00

### Photodynamic therapy using intravenous δ-aminolaevulinic acid-induced protoporphyrin IX sensitisation in experimental hepatic tumours in rats

K Svanberg[1,2], DL Liu[2,3], I Wang[1,2], S Andersson-Engels[2,4], U Stenram[2,5] and S Svanberg[2,4]

[1]*Department of Oncology, Lund University Hospital, S-221 85 Lund;* [2]*Lund University Medical Laser Centre, Lund University, S-221 85 Lund;* [3]*Department of Surgery, Lund University Hospital, S-221 85 Lund;* [4]*Department of Physics, Atomic Physics Division, Lund University, PO Box 118, S-221 00 Lund;* [5]*Department of Pathology, Lund University Hospital, S-221 85 Lund, Sweden.*

**Summary** The efficacy of photodynamic therapy (PDT) using δ-aminolaevulinic acid (ALA)-induced protoporphyrin IX (PpIX) sensitisation and laser light at 635 nm was investigated in the treatment of experimental hepatic tumours. The model of liver tumours was induced either by local inoculation or by administration of tumour cells through the portal vein in rats. ALA at a dose of 60 mg $kg^{-1}$ b.w. was intravenously administered 60 min before PDT. PpIX accumulation in tumour, normal liver and abdominal wall muscle was detected by means of laser-induced fluorescence (LIF). Laser Doppler imaging (LDI) was used to determine changes in the superficial blood flow in connection with PDT. Histopathological examinations were performed to evaluate the PDT effects on the tumour and the surrounding liver tissue, including pathological features in the microvascular system. The accumulation of PpIX, as monitored by LIF, showed high fluorescence intensities at about 635 nm in both the hepatic tumour tissue and normal liver and low values in the abdominal wall. LDI demonstrated that the blood flow in the treated tumour and its surrounding normal liver tissue decreased immediately after the PDT, indicating an effect on the vascular system. A large number of thrombi in the irradiated tumour were found microscopically 3 h after the PDT. The tumour growth rate showed a marked decrease when evaluated 3 and 6 days after the treatment. These results show that the ALA-PDT is effective in the inhibition of growth of experimental hepatic tumours.

**Keywords:** δ-aminolaevulinic acid; hepatic tumour; laser Doppler imaging; laser-induced fluorescence; photodynamic therapy; protoporphyrin IX; photosensitiser

Malignant tumours of the liver are considered to be highly therapy-resistant malignancies. Owing to a low resectability, the 5 year patient survival rate has not exceeded 12-18% (Adson 1986; Ringe *et al.*, 1991). Although various kinds of palliative treatment strategies, such as selective embolisation and ligation of the hepatic artery or the portal branch (Kawasaki *et al.*, 1994), laser-induced hyperthermia (Hahl *et al.*, 1990) and cryosurgery (Ravikumar *et al.*, 1991), have been employed clinically, the prognosis remains unsatisfactory. It has also been shown that chemotherapy and radiotherapy have a minor impact. Therefore, the development of new treatment strategies for this kind of tumour would be of great importance.

Photodynamic therapy (PDT) is a promising local treatment modality based on the selective accumulation of a photosensitiser in malignant tissue and the subsequent illumination with laser light of an appropriate wavelength. The laser light excites the molecules of the sensitiser. In an energy transfer process mediated by the excited sensitiser molecules, ground-state triplet oxygen in the tissue is excited to singlet oxygen. Singlet oxygen is known as a highly cytotoxic agent, causing membrane dysfunction at many cellular sites. The targets include the membrane of the cell, nucleus, mitochondrion, lysosome, Golgi apparatus and endoplasmic reticulum, as well as the endothelium of the microvascular system (Moan *et al.*, 1982; Henderson *et al.*, 1985).

During the past years, the role of PDT using intravenously administered haematoporphyrin derivative (HPD) has been investigated in the management of experimental hepatic tumour in rats (Pimestone *et al.*, 1982; Holt *et al.*, 1985; Nishiwaki *et al.*, 1989). When employing HPD-PDT in liver tumours it is important to consider that normal liver tissue accumulates HPD to a very high degree (Gomer and Dougherty, 1979; Bugelski *et al.*, 1981; Ankerst *et al.*, 1984; Svanberg *et al.*, 1986). For clinical use the dose-dependent transient skin sensitisation and the non-optimal light absorption profile have been considered as other obstacles. Second-generation photosensitisers, such as benzoporphyrin (BPD) (Richter *et al.*, 1987) and meso-tetrahydroxyphenylchlorin (Berenbaum *et al.*, 1986), with less pronounced skin sensitisation (especially for BPD) and more favourable light absorption profile, have been developed. The distribution of the substances in various organs and malignant tumour tissue has been experimentally investigated (Jamieson *et al.*, 1989; Andersson-Engels *et al.*, 1993; Alian *et al.*, 1994) and clinical phase I and II trials have been initiated (Ris *et al.*, 1991).

Recently, an alternative method of sensitisation was introduced by using the haem precursor δ-aminolaevulinic acid (ALA) (Kennedy *et al.*, 1990; Kennedy and Pottier, 1992). In the metabolic cycle of ALA, owing to variations in enzyme activity and bypassing of the feedback mechanism, an accumulation of the photodynamically very active protoporphyrin IX (PpIX) occurs in cells after excessive ALA administration locally, orally or intravenously. In particular, malignant tumour cells tend to synthesise more PpIX than non-malignant cells owing to a difference in the content of the enzymes regulating the haem cycle (Dailey and Smith, 1984; Leibovici *et al.*, 1988). Experimental work and clinical trials using ALA-induced PpIX sensitisation have been performed (Malik and Lugaci, 1987; Kennedy *et al.*, 1990; Kennedy and Pottier, 1992; Bedwell *et al.*, 1992; van Hillegersberg *et al.*, 1992*a,b*; Grant *et al.*, 1993; Svanberg *et al.*, 1994; Warloe *et al.*, 1994).

The aim of the present experimental study was to investigate the PpIX-mediated PDT effect on rat hepatic tumours and normal liver following intravenous injection of ALA. The distribution of PpIX before PDT was monitored by means of laser-induced fluorescence (LIF). The superficial blood flow in the tumour and its surrounding normal liver tissue before and after PDT was studied using a laser Doppler imaging (LDI) equipment. The histopathological changes were investigated 3 h, and 3 and 6 days after the

Correspondence: K Svanberg, Department of Oncology, Lund University Hospital, S-221 85 Lund, Sweden
Received 8 January 1996; revised 6 June 1996; accepted 19 June 1996

PDT. The PDT effects were evaluated macroscopically by calculation of the tumour growth rate 3 and 6 days after the PDT. Mechanisms involved in this therapeutic modality are discussed.

### Materials and methods

Two models for experimental hepatic tumours with local inoculation or administration of tumour cells through the portal vein were used. In total 40 inbred Wistar/Furth rats weighing 180–200 g were used for tumour induction. The cell line of the original colon adenocarcinoma was chemically induced by 1,2-dimethylhydrazine (Hedlund and Sjögren, 1980). In 30 rats $3 \times 10^5$ viable tumour cells were inoculated into the subcapsular region of the left lateral and the median lobes of the liver for induction of two primary tumours (Liu *et al.*, 1993). All 30 rats developed one tumour in each liver lobe with a mean diameter of $8 \pm 2$ mm 8 days after the inoculation. The tumour in the left lateral lobe was used for PDT and the other one served as an internal control tumour. The typical tumour positions in the rat liver are shown in Figure 1.

In ten rats $1 \times 10^6$ viable tumour cells were propagated into the liver through the portal vein for induction of secondary hepatic tumours. According to the experimental data reported by Lafreniere and Rosenberg (1986), 95% of all metastatic nodules within the parenchyma were found on the surface of the liver, thus counting only the surface nodules was thought to be a reliable method of evaluating the metastatic depositions. In this group four rats developed secondary tumours on the surface of the liver 20 days after the inoculation of tumour cells. The diameter of these tumours varied between 7 and 10 mm. One of the larger tumours in each rat liver was employed for PDT.

δ-Aminolaevulinic acid hydrochloride (Porphyrin Products, Logan, UT, USA; Lot no. 020592) at a dose of 60 mg $kg^{-1}$ b.w. was dissolved in approximately 2 ml of isotonic saline, and injected in all animals through the femoral vein 60 min before the laser irradiation. All rats were under general anaesthesia induced by chloral hydrate (250 mg $kg^{-1}$ b.w.) during the procedure.

**Figure 1** Schematic view of a rat liver with two inoculated tumours. Points for LIF measurements are identified in a scan through the tumour selected for treatment. Recordings from points E, F, and G are included in the tumour data and from A, B, C and I, J, K in the normal liver data. The full length of the scan is about 2–3 cm.

### *Laser-induced fluorescence*

The PpIX fluorescence following laser excitation was monitored in tumour and liver tissue in 15 rats, and in abdominal wall muscle in three rats. For the technique of fluorescence measurements, a fibre optic fluoresensor was used as described by Andersson-Engels *et al.* (1991, 1993). As an excitation source a nitrogen-laser pumped dye-laser was used, emitting light at 405 nm at a pulse repetition rate of 10 Hz. The full fluorescence spectrum was captured within the wavelength region 450–750 nm. The intensity of the free-standing fluorescence emission peak at 635 nm was evaluated, expressed in terms of an internal fluorescence standard. The tip of a 600 μm quartz fibre was put at the surface of the tumour and its surrounding normal liver tissue. In a scan across the region, typical measurement points were chosen as indicated in Figure 1. Fluorescence data were obtained for each tumour just before the PDT procedure.

### *Laser Doppler imaging measurements*

The superficial blood flow was monitored before and immediately after the PDT procedure using laser Doppler imaging equipment (Lisca Development, Linköping, Sweden). The light from a 3 mW He–Ne laser is reflected onto the tissue by an optical mirror system. The light beam (0.8 mm diameter) scans the object line by line, penetrating the tissue to a certain depth. In the presence of moving blood cells a fraction of the back-scattered and Doppler-broadened light is detected and converted into an electrical signal. The signal is processed to scale linearly with blood flow and is eventually used as an estimator of perfusion, i.e. the product of blood cell velocity and concentration in the sampling volume (Wårdell *et al.*, 1993).

### *Photodynamic therapy procedure*

Photodynamic therapy was performed 60 min after the injection of ALA, based on kinetic studies presented by Johansson *et al.* (1996). As a treatment laser a frequency-doubled Nd:YAG laser (Multilase 2500, Technomed International, Bron/Paris, France) pumping a dye laser (Multilase Dye 600, Technomed International) was used. The laser light at 635 nm was delivered in a single treatment with a total light dose of 100 J $cm^{-2}$. The light power density was kept below 110 mW $cm^{-2}$ in order to avoid local hyperthermia (in separate measurements it was checked that no significant temperature rise occurred). A border zone of 4 mm outside the tumour was included in the treatment field. The laser light was delivered perpendicularly to the area through an optical fibre followed by a microscope lens in order to obtain an even illumination of the whole area, by imaging the polished fibre end surface onto the treatment field. Without this arrangement the laser light intensity will be the highest in the centre with a gradual fall-off in light intensity towards the borders of the treated area.

Twenty-nine tumour-bearing rats (25 with primary tumour and four with secondary tumour) were treated with PDT (five animals died before PDT). For the follow-up examination the animals were divided into four groups. Groups I–III had primary local tumours and consisted of eight, ten and seven rats respectively. Group I was evaluated 3 h, group II 3 days and group III 6 days after the PDT. Group IV consisted of four animals with secondary tumours (portal vein tumour induction), which were evaluated 3 days after the PDT. In groups I–III the tumour in the left hepatic lobe was used for the PDT treatment and the other one located in the median lobe served as an internal control. In group IV one major tumour in each animal was chosen for the treatment.

Five rats in each of groups I–III were used to study the PpIX contents in the liver and tumour tissue. The PpIX signal in the abdominal wall was also measured in three rats in group III as mentioned before. The superficial blood flow was monitored in 17 rats in groups II, III and IV. Before the

PDT, the tumour size was measured with sliding calipers and the tumour volume was calculated using the formula $(a \times b^2) \times \pi/6$, ($a$ = maximum tumour diameter, $b$ = minimum tumour diameter) (Carlsson *et al.*, 1983). The individual tumour growth rate was calculated by forming the ratio betwen the tumour volume after and before PDT in groups II–IV. In groups II and III the tumour growth reduction factor was expressed as a ratio between the growth rate of the treated and non-treated tumours after and before PDT. A tumour growth reduction factor of 1 corresponds to natural growth, while a factor of 0.5 corresponds to only half of the tumour growth rate compared with the non-treated control tumour.

*Histological examination*

The treated and control tumours and livers in groups I–III and the treated ones in group IV were fixed in 4% formaldehyde and embedded in paraffin. Sections were stained with haematoxylin–eosin (HE) and examined under a microscope. The necrotic depth of the tumour and its surrounding normal liver was also measured by a pathologist using a microscope.

*Statistical analysis*

The mean value of tumour volume and tumour growth rate ± standard deviation (s.d.) was used to estimate therapeutic efficacy. Wilcoxon's test was used to evaluate the changes in tumour volume and growth rate at the different follow-up points. A $P$-value < 0.05 was considered statistically significant and $P < 0.01$ highly significant.

### Results

*Fluorescence investigation*

PpIX *in vivo* was monitored just before the PDT procedure. Three examples of *in vivo* fluorescence spectra recorded from tumour tissue, normal liver and abdominal wall for an injected rat are shown in Figure 2. The drug-specific dual-peaked fluorescence with the predominant peak at 635 nm is clearly seen in all three spectra, especially pronounced for the malignant tumour and liver tissue. The porphyrin signal exhibited variations within the larger tumours (diameter > 8 mm), while the smaller tumours showed a more even distribution. The liver tissue and abdominal wall showed almost no variations within the organ. The results of all fluorescence data from 15 rats (in total about 80 spectra) as evaluated in terms of an internal reference showed slightly higher fluorescence intensity in normal liver as compared with tumour tissue. The abdominal wall exhibited a low fluorescence intensity compared with the two other tissue types (Figure 3).

**Figure 2** *In vivo* fluorescence spectra recorded from an experimental hepatic tumour, normal liver tissue and abdominal wall muscle in a rat injected intravenously with 60 mg kg$^{-1}$ b.w. of ALA 60 min earlier. The ALA-induced PpIX-related fluorescence is characterised by a dual-peaked fluorescence with the predominant emission at 635 nm. The background-free intensity at 635 nm was evaluated.

*Photodynamic effects*

Immediately after the PDT procedure the surface of the whole irradiated field including the border zone outside the tumour was observed to be dark red and slightly swollen. The microscopic investigation at 3 h after the PDT (group I) revealed many fresh thrombi in the irradiated tumour tissue (Figures 4 and 5a), but no such changes were seen in the non-

**Figure 3** Diagram of background-free PpIX-related fluorescence intensity at 635 nm expressed in normalised units for experimental hepatic tumour, normal liver tissue and abdominal wall muscle. The data are recorded 60 min after i.v. injection of ALA (60 mg kg$^{-1}$ b.w.). The standard deviation indicated is high owing to variations between animals.

**Figure 4** A histopathological section of tumour tissue 3 h after the ALA-PDT. The laser light was delivered with a total light dose of 100 J cm$^{-2}$ and a fluence rate below the hyperthermic threshold (< 110 mW cm$^{-2}$). Several fresh thrombi (arrows) in the treated tumour can be seen (haematoxylin–eosin staining, HE × 115).

**Figure 5** (**a**) A fresh thrombus in the centre of the picture is seen in an irradiated tumour 3 h after the ALA-PDT (HE × 370). (**b**) A control tumour shows viable tumour cells and a vessel without thrombus (HE × 370).

irradiated control tumours (Figure 5b). Thrombi within necrotic tumour were found in all treated groups. No formation of thrombi in the surrouding irradiated normal liver was found.

The treated primary hepatic tumours in group II with a follow-up time of 3 days showed a marked reduction in the growth rate compared with the non-treated control tumours ($P<0.001$) (Figure 6a). The mean value of the ratio was $1.2\pm0.6$ (s.d.) (range 0.6–2.3) for the treated tumours and $3.2\pm1.5$ (s.d.) (range 1.7–6.0) for the non-treated ones. The ratio between the growth rate of the treated and non-treated tumours, the tumour growth reduction factor, showed a mean value of $0.42\pm0.22$ (s.d.) (range 0.15–0.8), illustrating a clear growth inhibition for the tumours treated with the PDT (Figure 6b). The microscopic investigation revealed necrosis with a maximum depth of 7 mm in the tumour area and a depth from 1.5–2 mm in the treated surrounding normal liver tissue 3 days after the PDT. The PDT-induced tumour necrosis in the liver tissue showed a clear demarcation towards the non-necrotic tissue (Figure 7). The macroscopic examination 3 days after the PDT procedure demonstrated a gross change in the texture of the tissue surface with well-demarcated necrosis in the treated area, including the surrounding liver tissue.

The histological examination in group III (6 days after PDT) showed an equally extensive necrosis in the surrounding normal liver tissue as well as a marked degeneration and necrosis of tumour cells as shown in group II. The tumour growth rate in group III showed a pronounced decrease in the treated tumours compared with the non-treated ones with the mean value of growth rate of $3.0\pm1.1$ (s.d.) (range 1.7–4.7) and $10.3\pm6.8$ (s.d.) (range 3.5–24) respectively ($P<0.02$) (Figure 8a). The tumour growth reduction factor showed the mean value of $0.36\pm0.22$ (s.d.) (range 0.12–0.63) (Figure 8b).

**Figure 6** Tumour growth rate for group II with a follow-up time of 3 days after the ALA-PDT. The individual tumour growth rate for the treated and non-treated tumour is expressed as the ratio of tumour volume before and 3 days after PDT (**a**). The threshold value at 1 is marked. A ratio below the threshold corresponds to a tumour volume reduction 3 days after the PDT. A tumour growth reduction factor, expressed as the ratio between the growth rate of the treated and non-treated tumour, is calculated for each individual animal (**b**). A tumour growth reduction factor of 1 corresponds to natural growth (no inhibition induced by PDT), a tumour growth reduction factor of 0.5 corresponds to only half of the growth rate compared with the non-treated control tumour.

Tumour regression was also observed in the group of secondary hepatic tumours (group IV). The histopathological examination also revealed a marked necrosis in the tumour and in the treated surrounding normal liver tissue 3 days after PDT. A clear decrease in the tumour growth reduction factor was achieved with the average ratio of $0.78\pm0.14$ (range 0.60–0.89).

After the PDT procedure an immediate decline of the blood flow was found in the irradiated tumour and tumour surrounding tissue (Figure 9). The mean perfusion value of the treated tumours decreased immediately from a relative value of $52\pm10$ before treatment to $22\pm8$ after treatment. The mean perfusion value of the treated surrounding normal liver tissue also decreased from $44\pm10$ before treatment to $16\pm8$ after treatment. The decreased blood flow lasted during

**Figure 7** Micrograph of a histopathological section of normal liver tissue 3 days after PDT. A marked boundary (arrows) between PDT-induced necrosis and non-affected tissue can be seen. The liver surface is at the top of the figure (HE × 150).

**Figure 8** Tumour growth rate for group III with a follow-up evaluation 6 days after PDT. The tumour growth rate for each individual is expressed as in Figure 6. The tumour growth reduction factor, calculated as in Figure 6, is also presented.

the follow-up periods of 3 and 6 days. Details of LDI results for more animals than studied here will be presented separately.

### Discussion

In this study PDT effects mediated by ALA-induced PpIX sensitisation were investigated in the management of experimental hepatic tumours. The synthesis of PpIX from the intravenously administered ALA was monitored by means of LIF. Our results show that 60 min after the ALA injection, the tumour and the surrounding normal liver tissue exhibit typical PpIX fluorescence with the dual-peaked fluorescence at 635 and 700 nm excited at 405 nm. The high PpIX signal in normal liver is caused by the fact that liver tissue has the highest capability for haem synthesis outside the haematopoietic system (Sardesi *et al.*, 1964). Also, the tumour tissue showed a remarkably high build-up of PpIX, with the tumour–liver ratio of 0.8 : 1 in our data. It should be noted that the induced tumour in this study is of non-hepatic origin. The high accumulation of PpIX in tumour tissue is facilitated by the altered activity of some of the enzymes regulating the haem cycle. The porphobilinogen deaminase exhibits an increased and ferrochelatase a decreased activity in some malignant tissue (Rubino and Rasetti, 1966; Schoenfeld *et al.*, 1988; Dailey and Smith, 1984; Leibovici *et al.*, 1988). The abdominal wall muscle showed a much lower PpIX fluorescence intensity with a ratio of 0.33 : 1 for the abdominal wall–liver and 0.42 : 1 for the abdominal wall–tumour.

The fluorescence intensity at 635 nm showed variations within the tumour, especially pronounced in larger tumours with a diameter >8 mm. A high level of protoporphyrin was detected in the peripheral tissue of the large tumour, which might be related to the specific hypervasculature of hepatic tumours. The central area in the larger tumours however, exhibited a much lower fluorescence intensity at 635 nm. This might be explained by the presence of an anoxic and necrotic part in the central area of the tumour. The normal liver tissue did not show any variation in the PpIX fluorescence. This variation in drug concentration is of central importance in the planning of PDT. LIF might thus be useful as a predictor of the PDT outcome, demarcating areas with less sensitiser and oxygen, two important parameters influencing the treatment efficacy. As LIF is a non-invasive method it can easily be employed in the preoperative planning of PDT with real-time measurements of the sensitiser. In the development of clinical PDT, it is of great importance in monitoring the sensitiser in connection with the treatment procedure. This is especially valuable in introducing new photosensitising agents for PDT, such as ALA, for which the best method of administration and the optimum time intervals for treatment

**Figure 9** Doppler blood perfusion images for a rat liver with two induced tumours, as illustrated in Figure 1. (**a**) High blood perfusion before the PDT. The line represents a section through the tumour to be treated for which perfusion values are plotted below. The arrows mark the tumour borders. (**b**) The same area after PDT of the chosen tumour with corresponding data.

have not yet been established. A further aspect is the possibility of using LIF for light dose measurements using the degree of bleaching of the sensitiser (Andersson *et al.*, 1992).

Henderson (1985) and Star (1986) have suggested that damage to the tumour vasculature and formation of thrombi in the irradiated tumour, resulting from early blood flow stasis in both arterioles and venules, with arteriolar vasoconstriction and thrombosis of venules and perivascular interstitial oedema, are important factors. Also, Pass (1991, 1993) suggested blood vessel damage as an important effect on tumour in the photochemical process. PDT may also induce the release of clotting factors and vasoactive intermediates that can increase blood coagulation and alter vessel tone in the treated tumour tissue (Evrard *et al.*, 1993; Pass, 1993). Injury of the vessel endothelium and blood cells caused by PDT is potentially another cause leading to vascular obliteration and formation of thrombi in the irradiated area.

The immediate reduction in the blood flow, as measured by LDI in this study, indicates a PDT effect on tumour vessels. This is in accordance with early studies and recent reports on vascular constriction in connection with PDT (Selman *et al.*, 1984; Kessel, 1984; Van der Veen *et al.*, 1994; Roberts *et al.*, 1994; Leveckis *et al.*, 1995). A sharply decreased blood flow was also noticed by a gross examination immediately after the irradiation, as the treated area became darker as a result of the treatment. The change in colour was especially pronounced in the tumour border area towards normal liver tissue, in which region the highest fluorescence intensity values were monitored. The surrounding normal liver tissue also exhibited the most pronounced blood flow decrease as measured by means of LDI (Figure 9). The histological investigation revealed a large number of fresh thrombi in the treated tumour tissue 3 h after PDT, indicating an immediate development of the blood shutdown. These histopathological changes are consistent with the results from the examination of LDI. The data from our study support the findings reported by Henderson *et al.* (1985) that PDT-induced destruction of tumour vessels is one of the major causes of secondary tumour cell death. Furthermore, the results from LDI also showed a decreased blood flow 3 and 6 days after the PDT treatment, which further verifies the above theory. The decreased blood flow following PDT after haematoporphyrin derivative or ALA systemic administration is in contrast to the increased blood flow observed after topical administration of ALA (Wang *et al.*, 1996). This indicates that different mechanisms are in action for the two administration modalities.

We have demonstrated a simultaneous increase of the PpIX signal in normal liver tissue and tumour tissue after the administration of ALA. The PpIX intensities were found to be modestly higher in the normal liver tissue than in tumour tissue. One may question whether the same damage develops in both tissue types treated with PDT. We did find a large number of thrombi after the PDT in the treated tumour vessels, including the treated secondary hepatic tumours, but not in the surrounding normal liver tissue.

Several possible explanations for this observation could be put forward. Firstly, it is well known that tumour vessels are tortuous and lack normal tissue structure; thus they are more susceptible to damage by the PDT treatment than normal liver tissue. Several investigators have documented the formation of platelet thrombi on the wall of treated vessels because PDT treatment can damage the basement membrane of vessels and endothelial cells (Henderson *et al.*, 1985). Secondly, coagulating factors, for example, thromboxane, are released from the treated tissue during PDT. These can induce tumour vessel constriction and blood aggregation followed by the formation of thrombi (Bellnier and Henderson, 1992). Thirdly, it has been reported that the blood flow in tumour tissue is slower than in normal liver tissue (Peterson, 1991), which is another reason for thrombi forming easily in tumour vessels.

Interestingly, the data from the present study indicate that the depth of necrosis following the PDT in the tumour tissue and normal liver does not correlate with the PpIX content. In other words, normal liver tissue showed a smaller depth of necrosis than did the treated tumour. We believe that this may be associated with the following factors. The colour of normal liver tissue is dark red, which leads to a strong absorption of the light, i.e. only superficial penetration of the light occurs during the PDT process resulting in a surface necrosis only. In contrast, the colour of rat liver tumour tissue is white or pale, leading to a reduced absorption of the light. The better distal penetration of the light causes a deeper necrosis in the treated tumour.

**Acknowledgements**
The skilful handling in tumour induction of Eva Gynnstam at the Wallenberg Laboratory as well as the talented statistical analysis performed by Jonas Ramstam are greatly appreciated. The work was supported by the Swedish Cancer Society, the Kamprad Foundation, the Swedish Board for Technical and Industrial Development and the Swedish Research Council of Engineering Sciences. We are also grateful to the Gunnar Nilsson Foundation for taking part in the financial support of DL Liu. I Wang is a Fellow of the Norwegian Cancer Foundation.

### References

ADSON MA (1986). Liver resection in primary and secondary liver cancer. In *Liver Surgery*. Bengmark S and Blumgart LH (eds). pp. 63-80. Churchill Livingstone: Edinburgh.

ALIAN W, ANDERSSON-ENGELS S, SVANBERG K AND SVANBERG S. (1994). Laser-induced fluorescence studies of *meso*-tetra(hydroxyphenyl)chlorin in malignant and normal tissue in rats. *Br. J. Cancer*, **70**, 880-885.

ANDERSSON T, BERG R, JOHANSSON D, KILLANDER D, SVANBERG K, SVANBERG S AND YANG YUANONG (1992). Photodynamic therapy in interplay with fluorescence diagnostics in the treatment of human superficial malignancies. *SPIE*, **1645**, 187-199.

ANDERSSON-ENGELS S, ELNER Å, JOHANSSON J, KARLSSON S-E, SALFORD LG, STRÖMBLAD L-G, SVANBERG K AND SVANBERG S. (1991). Clinical recording of laser-induced fluorescence spectra for evaluation of tumour demarcation feasibility in selected clinical specialities. *Lasers Med. Sci.*, **6**, 415-424.

ANDERSSON-ENGELS S, ANKERST J, JOHANSSON J, SVANBERG K AND SVANBERG S. (1993). Laser-induced fluorescence in malignant and normal tissue of rats injected with benzoporphyrin derivative. *Photochem. Photobiol.*, **57**, 978-983.

ANKERST J, MONTÁN S, SVANBERG K AND SVANBERG S. (1984). Laser-induced fluorescence studies of hematoporphyrin derivative (HPD) in normal and tumour tissue of rats. *Appl. Spectroscopy*, **38**, 890-896.

BELLNIER DA AND HENDERSON BW. (1992). Determinants for photodynamic tissue destruction. In *Photodynamic Therapy*. Henderson BW and Dougherty TJ (eds). pp. 117-127. Marcel Dekker: New York.

BEDWELL J, MACROBERT AJ, PHILLIPS D AND BOWN SG. (1992). Fluorescence distribution and photodynamic effect of ALA-induced PPIX in the DMH rat colonic tumour model. *Br. J. Cancer*, **65**, 818-824.

BERENBAUM MC, AKANDE SL, BONNETT R, KAUR H, IOANNOU S, WHITE RD AND WINFIELD UJ. (1986). *meso*-Tetra(hydroxyphenyl) porphyrins, a new class of potent tumour photosensitisers with favourble selectivity. *Br. J. Cancer*, **54**, 717-725.

BUGELSKI PJ, PORTER CW AND DOUGHERTY TJ. (1981). Autoradiographic distribution of hematoporphyrin derivative in normal and tumor tissue of the mouse. *Cancer Res.*, **41**, 4606-4612.

CARLSSON JR G, GULLBERG B AND HAFSTRÖM LO. (1983). Estimation of liver tumour volume using different formulas - an experimental study in rats. *J. Cancer Res. Clin. Oncol.*, **105**, 20-23.

DAILEY HA AND SMITH A. (1984). Differential interaction of porphyrins used in photoradiation therapy with ferrochelatase. *Biochem. J.*, **223**, 441-445.

EVRARD S, APRAHAMIAN M AND MARESCAUX J. (1993). Intra-abdominal photodynamic therapy: from theory to feasibility. *Br. J. Surg.*, **80**, 298-303.

GOMER CJ AND DOUGHERTY TJ. (1979). Determination of [$^3$H]- and [$^{14}$C]hematoporphyrin derivative distribution in malignant and normal tissue. *Cancer Res.*, **39**, 146-151.

GRANT WE, HOPPER C, MACROBERT AJ, SPEIGHT PM AND BOWN SG. (1993). Photodynamic therapy of oral cancer: photosensitisation with systemic aminolaevulinic acid. *Lancet*, **342**, 147-148.

HAHL J, HAAPIAINEN R, OVASKA J, PUOLAKKAINEN P AND SCHRÖDER T. (1990). Laser-induced hyperthermia in the treatment of liver tumors. *Lasers Surg. Med.*, **10**, 319-321.

HEDLUND G AND SJÖGREN HO. (1980). Induction of transplantation immunity to rat colon carcinoma isografts by implantation of intact fetal colon tissue. *Int. J. Cancer*, **26**, 71-73.

HENDERSON BW, WALDOW S, MANG TA, POTTER WR, MALONE PB AND DOUGHERTY TJ. (1985). Tumour destruction and kinetics of tumour cell death in two experimental mouse tumours following photodynamic therapy. *Cancer Res.*, **45**, 572-576.

HOLT S, TULIP J, HAMILTON D, CUMMINS J, FIELDS A AND DICK C. (1985). Experimental laser phototherapy of the Morris 7777 hepatoma in the rat. *Hepatology*, **5**, 175-180

JAMIESON CHM, DOLPHIN D AND LEVY J. (1989). Differential uptake of benzoprphyrin derivative (BPD) by leukemic versus normal cells. *SPIE Proc.*, **1065**, 152-163.

JOHANSSON J, BERG R, SVANBERG K AND SVANBERG S. (1996). Laser-induced studies of normal and malignant tumor tissue of rat following intravenous injection of $\delta$-amino levulinic acid. *Lasers Surg. Med.* (in press).

KAWASAKI S, MAKUUCHI M, KAKAZU T, MIYAGAWA S, TAKAYAMA T, KOSUGE T, SUGIHARA K AND MORIYA Y. (1994). Resection for multiple metastatic liver tumor after portal embolization. *Surgery*, **115**, 674-677.

KENNEDY JC, POTTIER RH AND PROSS DC. (1990). Photodynamic therapy with endogenous protoporphrin IX: basic principles and present clinical experience. *J. Photochem. Photobiol. B: Biol.*, **6**, 143-148.

KENNEDY JC AND POTTIER RH. (1992). Endogenous protoporphyrin IX, a clinically useful photosensitiser for photodynamic therapy. *J. Photochem. Photobiol. B: Biol.*, **14**, 275-292.

KESSEL D. (1984). Hematoporphyrin and HPD: photophysics, photochemistry and phototherapy. *Photochem. Photobiol.*, **39**, 851-859.

LAFRENIERE R AND ROSENBERG SA. (1986). A novel approach to the generation and identification of experimental hepatic metastases in a murine model. *J. Natl Cancer Inst.*, **76**, 309-315.

LEIBOVICI L, SCHOENFELD NILI, YEHOSHUA HA, MAMET R, RAKOWSKI E, SHINDEL A AND ATSMON A. (1988). Activity of porphobilinogen deaminase in peripheral blood mononuclear cells of patients with metastatic cancer. *Cancer*, **62**, 2297-2300.

LEVECKIS J, BROWN NJ AND REED MWR. (1995). The effect of aminolaevulinic acid-induced, protoporphyrin IX-mediated photodynamic therapy on the cremaster muscle microcirculation *in vivo*. *Br. J. Cancer*, **72**, 1113-1119.

LIU LX, SVANBERG K, WANG I, STENRAM U, ANDERSSON-ENGELS S AND SVANBERG S. (1993). Liver twin tumours: a new experimental hepatic tumour model in the investigation of various treatment strategies. *Med. Sci. Res.*, **21**, 703-706.

MALIK Z AND LUGACI H. (1987). Destruction of erythroleukaemic cells by photoactivation of endogenous porphyrin. *Br. J. Cancer*, **56**, 589-595.

MOAN J, JOHANNESSEN JV AND CHRISTENSEN T. (1982). Porphyrin-sensitized photoinactivation of human cells *in vitro*. *Am. J. Pathol.*, **109**, 184-192.

NISHIWAKI Y, NAKAMURA S AND SAKAGUCHI S. (1989). New method of photosensitizer accumulation for photodynamic therapy in an experimental liver tumour. *Lasers Surg. Med.*, **9**, 254-263.

PASS HI. (1991). Photodynamic therapy for lung cancer. *Chest Surg. Clin. North Am.*, **1**, 135-151.

PASS HI. (1993). Photodynamic therapy in oncology: mechanisms and clinical use. *J. Natl Cancer Inst.*, **85**, 443-456.

PETERSON HI. (1991). Modification of tumour blood flow - a review. *Int. J. Radiat. Biol.*, **60**, 201-210.

PIMSTONE NR, HORNER IJ, SHAYLOR-BILLINGS J AND GANDHI SN. (1982). Hematoporphyrin-augmented phototherapy in experimental liver cancer in the rat. *SPIE Proc.*, **357**, 60-67.

RAVIKUMAR TS, KANE R, CADY B, JENKINS R, CLOUSEL Y AND STEELE Jr G. (1991). A 5-year study of cryosurgery in the treatment of liver tumours. *Arch. Surg.*, **126**, 1520-1524.

RICHTER AM, KELLY B, CHOW DJ, LIU J, NEIL TOWERS GH, DOLPHIN D AND LEVY J. (1987). Preliminary studies on a more effective phototoxic agent than hematoporphyrin. *J. Natl Cancer Inst.*, **79**, 1327-1332.

RINGE B, PICHLMAYR R, WITTEKIND C AND TUSCH G. (1991). Surgical treatment of hepatocellular carcinoma: experience with liver resection and transplantation in 198 patients. *World J. Surg.*, **15**, 270-285.

RIS H-B, ALTERMATT HJ, INDERBITZI R, HESS R, NACHBUR B, STEWART JCM, WANG Q, LIM CK, BONNETT R, BERENBAUM MC AND ALTHAUS U. (1991). Photodynamic therapy with chlorines for diffuse malignant mesothelioma: initial clinical results. *Br. J. Cancer*, **64**, 1116-1120.

ROBERTS DJH, CAIRNDUFF F, DRIVER I, DIXON B AND BROWN SB. (1994). Tumour vascular shutdown following photodynamic therapy based on polyhaematoporphyrin or 5-aminolevulinic acid. *Int. J. Oncol.*, **5**, 763–768.

RUBINO GF AND RASETTI L. (1966). Porphyrin metabolism in human neoplastic tissue. *Panminerva Med.*, **8**, 290–292.

SARDESAI VM, WALDMAN J AND ORTEN JM. (1964). A comparative study of porphyrin biosynethesis in different tissues. *Blood*, **24**, 178–186.

SCHOENFELD N, EPSTEIN O, LAHAV M, MAMET R, SHARKLAI M AND ATSMON A. (1988). The heme biosynthetic pathway in lymphocytes of patient with malignant lymphoproliferative disorders. *Cancer Lett.*, **43**, 43–48.

SELMAN SH, KREIMER-BIRNBAUM M, KLAUNIG JE, GOLDBLATT PJ, KECK RW AND BRITTON SL. (1984). Blood flow in transplantable bladder tumors treated with hematoporphyrin derivative and light. *Cancer Res.*, **44**, 1924–1927.

STAR WM, MARUNISSEN HPA, BERG-BLOCK AE, VERSTEEG AC, FRANKEN KAP AND REINHOLD HS. (1986). Destruction of rat mammary tumour and normal tissue microcirculation by haematoporphyrin derivative photoradiation observed *in vivo* in sandwich observation chambers. *Cancer Res.*, **46**, 2532–2540.

SVANBERG K, KJELLÉN E, MONTÁN S, SJÖHOLM E AND SVANBERG S. (1986). Fluorescence studies of hematoporphyrin derivative in normal and malignant rat tissue. *Cancer Res.*, **46**, 3803–3808.

SVANBERG K, ANDERSSON T, KILLANDER D, STENRAM U, ANDERSSON-ENGELS S, BERG R, JOHANSSON J AND SVANBERG S. (1994). Photodynamic therapy of non-melanoma malignant tumours of the skin topical $\delta$-amino levulinic acid (ALA) sensitisation and laser irradiation. *Br. J. Dermatol.*, **130**, 743–751.

VAN DER VEEN N, VAN LEENGOED HLLM AND STAR WM. (1994). *In vivo* fluorescence kinetics and photodynamic therapy using 5-aminolevulinic acid-induced porphyrin: increased damage after multiple irradiations. *Br. J. Cancer.*, **70**, 867–872.

VAN HILLEGERSBERG R, MARIJINISSEN JPA, KORT WJ, ZONDERVAN PE, TERPSTRA OT AND STAR WM. (1992*a*). Interstitial photodynamic therapy in a rat liver metastasis model. *Br. J. Cancer*, **66**, 1005–1014.

VAN HILLEGERSBERG R, VAN DEN BERG JWO, KORT WJ, TERPSTRA OT AND WILSON JH. (1992*b*). Selective accumulation of endogenously produced porphyrins in a liver metastasis model in rats. *Gastroenterology*, **103**, 647–651.

WANG I, ANDERSSON-ENGELS S, NILSSON GE, WÅRDELL K AND SVANBERG K. (1996). Superficial bloodflow following photodynamic therapy of malignant skin tumours measured by laser Doppler perfusion imaging. *Br. J. Dermatol.*, (in press).

WARLOE T, PENG Q, HEYERDAHL H, MOAN J, STEEN HB AND GIERCKSKY K-E. (1994). Photodynamic therapy with 5-aminolevulinic acid induced porphyrins and DMSO/EDTA for basal cell carcinoma. *SPIE*, **2371**, 226–235.

WÅRDELL K, JAKOBSSON A AND NILSSON GE. (1993). Laser Doppler perfusion imaging by dynamic light scattering. *IEEE Trans. Biomed. Eng.*, **40**, 309–316.